# Cross-National Comparison of Hearing Status, Social Activities, and Verbal Memory: United States, Mexico and China

**DOI:** 10.1093/geroni/igaf122.3251

**Published:** 2025-12-31

**Authors:** Chengming Han, Brian Downer, Rebeca Wong, Zhiwei Hu

**Affiliations:** UTMB Health, The University of Texas Medical Branch, Galveston, Texas, United States; UTMB, Galveston, Texas, United States; The University of Texas Health Science Center at San Antonio, San Antonio, Texas, United States; the University of Texas Medical Branch, Galveston, Texas, United States

## Abstract

**Objective:**

This study explores the mediating effect of social activities on the association between hearing status and cognitive function across three countries.

**Method:**

Data from three waves of the HRS (United States), MHAS (Mexico), and CHARLS (China), collected from 2011 to 2021, were analyzed. Multilevel models were used to examine the association between self-reported hearing status (fair/poor vs. good/excellent) and verbal memory. The mediating effects of social activities were assessed using the Karlson-Holm-Breen (KHB) method.

**Results:**

Fair/poor hearing was associated with lower verbal memory in the HRS (b=-0.145, SE = 0.023) and CHARLS (b=-0.056, SE = 0.020), but not in MHAS (b=-0.001, SE = 0.018). Social activities were positively associated with verbal memory in all three datasets. Fair/poor hearing was associated with reduced social activities in the HRS (OR = 0.838, p < 0.001), MHAS (OR = 0.847, p < 0.01), and CHARLS (OR = 0.779, p < 0.001). The association between self-rated hearing and verbal memory remained statistically significant after adjusting for social activities in the HRS (b=-0.144, SE = 0.023) and CHARLS (b=-0.045, SE = 0.021).

**Conclusion:**

We did not find universal mediation effects of social activities on the association between hearing status and verbal memory. These findings underscore the need for context-specific approaches to addressing sensory loss and cognitive health.

